# Supplementary material for: Has the COVID-19 Pandemic Led to Changes in the Tasks of the Primary Care Workforce? An International Survey among General Practices in 38 Countries (PRICOV-19)
Source: Int J Environ Res Public Health. 2022 Nov 20;19(22):15329. doi: 10.3390/ijerph192215329 (PMC9690243; doi:10.3390/ijerph192215329)
Supplement: Supplementary file 1 [file ijerph-19-15329-s001.zip › ijerph-1944432-supplementary.pdf]

Supplementary tables

Supplementary table S1: Scale on task changes: average, median and 25th and 75th percentile by country.

Country	Average	Median	P-25	P-75
Austria	2,74	2,81	2,55	3,02
Belgium	2,75	2,77	2,43	3,09
Bosnia and Herzegovina	2,15	1,96	1,72	2,72
Bulgaria	2,95	2,97	2,73	3,19
Croatia	2,92	2,99	2,73	3,40
Czech Republic	2,95	3,00	2,80	3,26
Cyprus	3,05	3,11	2,79	3,33
Denmark	2,94	2,87	2,60	3,29
Estonia	2,97	2,99	2,75	3,26
Finland	2,00	2,00	1,74	2,26
France	2,58	2,67	2,25	3,02
Germany	2,90	2,90	2,67	3,19
Greece	2,89	2,95	2,61	3,09
Hungary	2,98	3,01	2,77	3,28
Iceland	2,73	2,74	2,46	3,15
Ireland	2,82	2,85	2,59	3,08
Israel	2,26	2,22	1,91	2,70
Italy	3,46	3,50	3,24	3,76
Kosovo*	3,03	3,08	2,73	3,43
Latvia	3,40	3,66	3,34	3,80
Lithuania	3,22	3,32	2,90	3,55
Luxembourg	2,69	2,79	2,29	3,25
Malta	2,30	2,55	1,90	2,69
Moldavia	3,28	3,22	3,04	3,64
The Netherlands	2,75	2,82	2,43	3,17
North Macedonia	2,59	2,85	2,38	2,89
Norway	2,67	2,72	2,38	2,94
Poland	3,08	3,09	2,86	3,50
Portugal	3,11	3,10	2,83	3,51
Romania	3,34	3,47	3,05	3,83
Serbia	2,40	2,67	1,93	2,90
Slovenia	2,75	2,79	2,48	3,08
Spain	2,76	2,82	2,44	3,11
Sweden	2,83	2,90	2,56	3,12
Switzerland	2,87	2,85	2,65	3,25
Turkey	2,51	2,62	2,09	3,03
Ukraine	2,79	2,93	2,53	3,03
The United Kingdom	2,71	2,72	2,39	3,00

Supplementary table S2: Number of patients, number of GPs and GP trainees, and total number of paid staff by country (average and median)

	Practice size (patients)		Number of GPs and trainees		Total number of staff		
Country	Average	Median	Average	Median	Average	Median	N between
Austria	2,468	1,600	1.8	1	7.0	5	137-140
Belgium	3,118	2,500	2.6	2	5.5	3.0	286-478
Bosnia and Herzegovina	6,976	5,000	3.0	3	7.8	6	38-40
Bulgaria	2,314	1,745	11.3	1	3.2	2	102
Croatia	1,707	1,742	1.1	1	2.3	2	147-149
Czech Rep	2,050	1,920	1.9	1	3.7	3	110-111
Cyprus	2,541	2,463	1.9	2.0	6.1	4.0	11-13
Denmark	4,418	3,300	3.0	3	7.8	8	39
Estonia	4,420	2,441	3.2	2	10.3	7	117-118
Finland	3,601	1,500	8.1	5	57.2	40	97-115
France	4,149	2,650	5.5	3	8.7	5	614-641
Germany	5,452	3,000	2.6	2	10.4	8.5	246-262
Greece	24,406	15,000	7.7	6	27.2	25	85-94
Hungary	1,855	1,750	1.2	1	2.7	2	222-224
Iceland	11,636	12,900	6.7	8	29.1	30	30-31
Ireland	6,022	4,500	3.7	3	10.6	9	182-185
Israel	4,014	3,200	3.2	3	16.1	12	87-88
Italy	3,952	3,001	2.8	2	4.0	3	206-207
Kosovo*	22,354	12,000	8.5	5.5	81.3	50	47-74
Latvia	1,951	1,850	1.1	1	3.8	3	148-149
Lithuania	13,653	9,100	10.5	7.5	134.5	40	50-53
Luxemburg	6,480	3,000	3.6	3.0	7.2	5.0	17-20
Malta	17,667	3,500	9.5	1	12.1	5	11-13
Moldova	19,140	8,048	13.8	4	87.3	29	72
Netherlands	4,436	3,200	3.4	2	11.9	10	165-166
North Macedonia	2,478	2,132	1.6	1.0	3.9	3.0	46
Norway	4,908	4,500	5.3	5	10.5	10	144
Poland	6,141	4,525	3.5	3	12.5	10	206-211
Portugal	12,816	12,000	7.7	7	24.8	23	222-225
Romania	2,037	1,918	1.4	1	2.9	3	99-101
Serbia	25,830	10,000	14.4	10	44.2	25.5	102-113
Slovenia	1,762	1,800	1.3	1	3.3	3	189-192
Spain	17,531	17,000	12.7	12	42.0	39	299-301
Sweden	11,057	10,100	6.0	5	45.8	40	84-86
Switzerland	5,958	4,000	3.1	2	11.3	8	83-89
Turkey	13,081	10,750	4.3	4	10.3	9	142-144
Ukraine	21,248	13,000	10.5	6.0	59.0	25.0	244-256
United Kingdom	7,804	7,450	6.1	6.0	24.7	25.0	23-24

Supplementary table S3: Country and health system characteristics *

Country	*COVID-19* Confirmed cases 1st wave	*Urgency* Deaths 1st wave	*Per million* Confirmed cases 3 months before survey	*inhabitants* Deaths 3 months before survey	*Role of GPs*	*task shifting around 2011*	*Strength of PC*	*Nurse prescrip tion rights*
Austria	1781	69	33,674	729	5	0.95	2.24	N
Belgium	4,698	770	42,284	580	7	0.44	2.23	N
Bosnia and Herzegovina	685	39	20,735	632	4	-	-	
Bulgaria	310	15	23,878	1,123	6	1.78	2.14	N
Croatia	544	23	27,349	898	7	-	-	
Cyprus	1,012	19	27,199	204	3	2.29	1.97	Y
Czech Rep	784	28	46,452	734	6	2.28	2.16	N
Denmark	1,856	92	22,233	261	3	3.45	2.39	Y
Estonia	1,333	48	7,480	41	6	3.79	2.30	Y
Finland	1,122	53	3,023	11	7	3.70	2.31	Y
France	2,686	408	22,642	488	7	-	2.17	Y
Germany	2,089	94	20,039	556	4	2.56	2.22	N
Greece	271	15	11,568	429	6	1.50	2.12	N
Hungary	355	46	42,623	1,553	7	3.15	2.10	N
Iceland	4,886	27	3,015	46	5	2.72	1.84	N
Ireland	4,808	305	27,001	279	4	3.39	2.18	Y
Israel	1,788	29	23,467	205	2	-	-	
Italy	3,709	524	29,648	633	4	0.62	2.34	N
Kosovo*	530	16	19,909	396	5	-	-	
Latvia	520	10	20,909	320	6	3.49	2.17	N
Lithuania	565	15	62,027	986	5	3.27	2.28	N
Luxemburg	6,180	164	11,537	27	6	0.19	1.94	N
Malta	1,031	12	22,711	393	4	1.71	2.14	N
Moldova	1428	50	20,681	407	7	-	-	
Netherlands	2,548	329	36,082	383	2	3.69	2.49	Y
North Macedonia	835	47	28,624	955	-	2.70	2.24	N
Norway	1504	42	6,297	53	7	2.52	2.27	Y
Poland	477	24	26,879	637	3.5	2.96	2.14	Y
Portugal	2,811	117	33,171	485	7	3.72	2.41	N
Romania	859	56	20,220	400	7	2.89	2.31	N
Serbia	1,519	33	50,478	465	7	-	-	N
Slovenia	705	50	35,065	626	5	2.74	2.37	N
Spain	4,924	587	33,318	480	7	3.76	2.43	Y
Sweden	2,963	359	39,030	605	5	3.96	2.25	Y
Switzerland	3,501	203	25,708	557	4	1.47	2.05	Y

Turkey	1,722	48	23,901	172	4	2.88	2.28	N
Ukraine	399	11	14,452	346	-	-	-	
United Kingdom	3,322	489	8,521	307	3	3.95	2.51	Y

* Source and definition of these variables are in the Methods section under 'measurements'.

Supplementary table S4: Multilevel analysis task changes in GPs, trainees and practice staff; sensitivity analysis with only full data

	Model 1: empty model Coefficient (SE)	Model 2: practice variables Coefficient (SE)	Model 3: interaction terms Coefficient (SE)	Model 4: Country variables [a] Coefficient (SE)
Fixed part				
Constant	2.824 (0.057)	2.505 (0.076)	2.517 (0.076)	
Staff absence		-0.0001 (0.0009)	-0.004 (0.002)*	
coped with internally		-0.004 (0.008)	-0.013 (0.008)	
coped with neighbouring practices		-0.032 (0.010)*	-0.029 (0.010)**	
improved cooperation with neighbouring practices		0.071 (0.010)**	0.072 (0.010)**	
GPS happy with the task shifting		0.060 (0.009)**	0.059 (0.009)**	
GPs don't feel prepared		0.016 (0.010)	0.016 (0.010)	
need further training		0.018 (0.010)	0.018 (0.010)	
Practice size		-4.277e-008 (1.34e-006)	-2.731e-008 (1.362e-006)	
Number of GPs and trainees		-0.0004 (0.0005)	-0.0006 (0.0005)	
Total number of paid staff		-4.792e-005 (0.003)	0.001 (0.002)	
Number of disciplines		0.003 (0.005)	0.004 (0.005)	
GPs paid (mixed) fee-for-service		-0.033 (0.032)	-0.034 (0.032)	
Practice location (ref. big city)				
- suburbs		-0.028 (0.034)	-0.027 (0.034)	
- (small) towns		-0.024 (0.028)	0.027 (0.028)	
- mixed urban-rural		0.022 (0.027)	0.023 (0.027)	

- rural		-0.032 (0.029)	0.032 (0.029)	
Practice population elderly/chronic conditions		0.022 (0.008)*	0.021 (0.008)*	
Practice population other vulnerable populations		0.002 (0.004)	0.003 (0.004)	
Interaction staff absence*coping internally			0.002 (0.0006)**	
Interaction staff absence*coping neighbouring practices			0 (0)	
Interaction staff absence*coping improved cooperation			-0.001 (0.001)	
COVID-19 cases per million population during 1st wave				0.009 (0.005) (p=0.09)
Idem COVID-19 mortality				-0.003 (0.005)
COVID-19 cases per million population 3 mnths before survey				-0.0002 (0.005)
Idem COVID-19 mortality				0.006 (0.005)
Role of GPs during pandemic				0.034 (0.038)
Strength of PC				-0.073 (0.428)
Nurse prescribing rights (yes)				-0.154 (0.128)
Degree of task shifting in 2012				-0.025 (0.063)
Random part				
Country variance	0.11 (0.028)	0.12 (0.029)	0.12 (0.029)	
Practice variance	0.282 (0.007)	0.27 (0.007)	0.27 (0.007)	
ICC (%)	28.5	29.9	29.9	

* p<0.05
** p<0.01
[a] Country variables have been added one by one; coefficients of practice level variables are not reported – they differ only marginally from those in Model 3.
